# Combined Edge- and Stixel-based Object Detection in 3D Point Cloud

**DOI:** 10.3390/s19204423

**Published:** 2019-10-12

**Authors:** Fangchao Hu, Dong Yang, Yinguo Li

**Affiliations:** 1 Department of Computer Science and Technology, Chongqing University of Posts and Telecommunications, Chongqing 400065, China; 2 Department of Automation, Chongqing University of Posts and Telecommunications, Chongqing 400065, China; s170331069@stu.cqupt.edu.cn (D.Y.); liyg@cqupt.edu.cn (Y.L.)

**Keywords:** autonomous vehicle, objects’ edge detection, stixel histograms accumulate, point cloud segmentation

## Abstract

Environment perception is critical for feasible path planning and safe driving for autonomous vehicles. Perception devices, such as camera, LiDAR (Light Detection and Ranging), IMU (Inertial Measurement Unit), etc., only provide raw sensing data with no identification of vital objects, which is insufficient for autonomous vehicles to perform safe and efficient self-driving operations. This study proposes an improved edge-oriented segmentation-based method to detect the objects from the sensed three-dimensional (3D) point cloud. The improved edge-oriented segmentation-based method consists of three main steps: First, the bounding areas of objects are identified by edge detection and stixel estimation in corresponding two-dimensional (2D) images taken by a stereo camera. Second, 3D sparse point clouds of objects are reconstructed in bounding areas. Finally, the dense point clouds of objects are segmented by matching the 3D sparse point clouds of objects with the whole scene point cloud. After comparison with the existing methods of segmentation, the experimental results demonstrate that the proposed edge-oriented segmentation method improves the precision of 3D point cloud segmentation, and that the objects can be segmented accurately. Meanwhile, the visualization of output data in advanced driving assistance systems (ADAS) can be greatly facilitated due to the decrease in computational time and the decrease in the number of points in the object’s point cloud.

## 1. Introduction

To securely and efficiently drive in increasingly complex traffic, the drivers must have a distinct and correct understanding of the environment. Nevertheless, obtaining and processing driving-related information is a great challenge due to the complexity of the environment and evolving traffic dynamics. For instance, complex architecture (e.g., the flyovers, switchback, abrupt slope), moving pedestrians and vehicles in an urban area, dim illumination in underground parking lots, feeble GPS signal, and inaccurate positioning increases the difficulty of driving [1,2]. Such challenges exist not only for human drivers, but also for autonomous vehicles whose safety heavily relies on knowledge of the surrounding environment.

To address these issues, many vehicle manufacturers focus on developing the advanced driving assistance system (ADAS) to assist drivers with decision making [3]. It plays a more and more important role to ensure safety nowadays with advanced technology. The most common facilities for perception on autonomous vehicles are radars, LiDAR (Light Detection and Ranging), cameras, GPS (Global Positioning System), and INS (Inertial Measurement Unit) [4]. LiDAR, which is equipped in Google self-driving cars to identify objects and obstacles during driving, can be utilized to sense the driving environment extremely efficiently and precisely. However, LiDAR cannot be widely employed in ordinary vehicles due to its stiff price. While LiDAR can directly provide important three-dimensional (3D) information, the required data lack rich appearance. This is where a camera-based system bears high potential [5]. By comparison, the stereo camera is capable of achieving similar functions while maintaining low operational costs. The stereo camera has several advantages in practice. First, it is able to obtain more real-time environmental data for driving, due to the short work cycle. Second, the number of 3D points that is produced by LiDAR is more redundant than that of the stereo camera. Therefore, this paper exploits the stereo camera-based method, which reduces the computation burden of image processing to achieve real-time driving assistance.

This paper attempts to identify obstacles in the driving environment, which is the most essential thing for driving. Numerous algorithms have been proposed for obstacle segmenting in different applications. Yu et al. [6] proposed a graph matching-based scene parsing framework to segment 3D point cloud. The graph matching approach can effectively interpret the street scene, but it is computationally expensive as it works on a graph processing at the voxel-wise level. Liu et al. [7] developed a four-step method to label 3D point cloud. This method enhances the inference accuracy by transferring the reliable two-dimensional (2D) labeling results into 3D. However, it demands a huge amount of points that are obtained from a 3D scanner, and significantly adds to the computational burden. Xiao et al. [8] utilized mobile laser scanning (MLS) data to detect street-side vehicles and classify the type of vehicles. This method improves the recognition rate and the localization precision. The laser scanner produces many points to process, and it costs a lot of time and computational resources. Zhang et al. [9] deployed 2D convolutions to segment 3D point cloud. It was faster and required less memory, but it requires pre-training. Narksri et al. [10] utilized the geometric characteristics of point cloud to primarily segment point cloud. Barea et al. [11] deployed the RGB-based CNN and projected into LiDAR point cloud to segment point cloud. Pan et al. [12] presented a top-down method for segmenting main bridge components, combined with rule-based classification, to produce a labeled 3D model from point cloud. Huang et al. [13] represented a multi-scale feature to classify the point cloud into a more effective and discriminative performance. Pan et al. [14] proposed a graph-cut-based method to segment point clouds automatically from multi-view images. This method does not require manual labeling of points and can be automatically run. Moreover, some object proposal methods are proposed to improve the performance of segmentation. For example, Sun et al. [15] proposed a multi-scale approach to detect a vehicle’s edges using three different image resolutions. Betke [16] suggested a coarse-to-fine search technique, in which the coarse search identified groups of prominent edges and a finer search performed on these regions detected rectangular-shaped objects. However, with the stereo camera-based method, the 3D points are generated from images with an indirect method, and the precision of localization of geometry extraction and 3D modeling are still challenging tasks which remain unresolved. 

There are lots of researches on 3D scene segmentation, many of which deal with 3D point cloud directly, rather than the abovementioned indirect method. Because 3D point clouds contain essential information of shape and spatial, which can characterize the contextual and spatial relationships between different objects, these characteristics provide the cues to segment the objects in 3D point cloud [17]. On the other hand, compared with the objects in indoor environments, the characteristics of objects in outdoor environments are dissimilar, e.g., the objects are apparently larger and the influences of illumination are stronger [18]. Besides, the speed of motion of objects outdoors are faster. These characteristics result in objects that are difficult to recognize and segment. 

In this paper, we focus on edge detecting-based object segmentation in 3D point cloud scenes. Specifically, we aim to address the problem of object segmentation in 3D scenes with unsupervised learning by using the stixel estimation and edges of objects extracted from sequential 2D corresponding images. The unsupervised learning method has lower computation complexity than the supervised learning method, especially the deep learning method which cannot run on a vehicle-mounted computing element. Besides, the unsupervised learning method can avoid system error, which is caused by a lack of classifying. It should be noted that the proposed method works in the scenario where a whole scene point cloud is merged from multi-view point clouds. The steps of the proposed method in this paper are stated as follows. First, edges of the objects in corresponding images, which are acquired by the stereo camera, are detected respectively. Second, the feature points in images are detected in the detected area of the edges. Third, the feature points are projected into the 3D scene and match with the whole scene point cloud. Finally, we segment the matched points from the whole scene point cloud. The proposed method utilizes few feature points in the detected area of edges to match and segment the whole scene point cloud, rather than segmenting the whole scene point cloud directly. The unsupervised learning method can be easily implemented in autonomous vehicles; the double threshold includes edge-based and stixel-based estimations which are more accurate in object proposal. Furthermore, the local reconstructed point clouds of objects are efficient to register and match with whole scene point clouds. The points that need to be matched are reduced. We partitioned the object point cloud from whole scene point cloud instead of segmenting the point cloud point-wisely. It implements real-time 3D object detection for autonomous vehicles.

This paper is organized as follows. Section 2 reviews the related work in segmentation and scene analysis. The proposed method is explicitly presented in Section 3. Section 4 and Section 5 discuss the experimental results and comparisons with other existing methods. Finally, we draw a conclusion of this paper in Section 6.

## 2. Related Work

A stereo camera is a cost-effective device and can efficiently perceive the driving environment. Thereby, it is extensively selected to mount on the autonomous vehicle by many vehicle manufacturers and research institutions [19,20]. The interested objects can be segmented and tracked effectively by the depth information of the stereo camera or the fusion information with monocular cues. Depth information enables robust feature tracking over a long distance. Recently, there has been an increasing research interest in semantic segmentation of 3D point cloud [21]. It is applicable for both indoor and outdoor environments and has corresponding tasks, such as the segmentation of tables, desks, trees, cars, roads, and pedestrians. Hitherto, most robotic scene understanding works have focused on 3D indoor scenes, and the technologies have become ripened with the development over the decades. Related applications such as mapping and autonomous driving can be deployed in the well-studied method of indoor environments. In this paper, the task is to segment point clouds from whole scene point clouds into a few dense object point clouds [22]. Outdoor scenes have more challenges, because they are covered by more extensive objects. A major challenge of this task has arisen from the fact that urban transport scenes of great majority are disordered and obstructed. Although significant progress has been made in the existing study of object segmentation of large architectural elements (e.g., walls, road edges), the performance is still far from satisfactory for on-road objects such as vehicles and pedestrians.

With the evolution of 3D reconstruction techniques such as parallel tracking and mapping (PTAM) [23], dense tracking and mapping (DTAM) [24], and Kinect fusion [25], a potential solution is to exploit the merged point cloud, which can achieve high performance for indoor scenes where laser data are classified and modeled into a few rough geometric feasibilities to label the point cloud of indoor scenes. The other solutions implement segmentation and detection on individual frames, respectively, and the outputs are merged into a point cloud and undergo a joint optimization procedure. Most of these approaches use features that describe local shapes and appearances to segment individual 3D laser points or voxels, and through the inference of graphical model, the joint optimization is typically accomplished by spatial and temporal smoothing.

The most related work to ours is presented in [14]. The authors utilized a weighted graph whose nodes represent points and edges that connect each point to its k-nearest neighbors. Next, after refining the initial segmentation, GMM (Gaussian Mixture Model) are created from the color and density features of points in object and background classes, respectively. The downside of their graph-cut segmentation approach is the spoil of object boundaries, leading to the misclassification of objects. In this paper, in order to maintain accurate object boundaries, we present a detection-based scheme, in combination with edge detection of 2D image pairs, to segment the 3D whole scene point clouds. First, we combine the edge of objects and stixel histograms in corresponding images to obtain a boundary region of the objects as the initial distribution. Second, we reconstruct the 3D sparse point cloud of objects based on the boundary region. Third, reconstructed points are projected to 3D coordinate fit with the whole scene point cloud. Finally, we segment the point cloud to obtain the objects which are represented in 3D dense point cloud.

The contribution of the proposed method is twofold. First, we utilize the edge of objects and stixel histograms to find the object proposal area, reduce the computation time, and find the objects without training or classification. Second, the proposed method simplifies the procedure of object location, and improves the positioning accuracy. At the stage of sparse point cloud matching with dense point cloud, less points will be matched with the current point cloud. It not only improves the accuracy of partition, but also reduces the time cost of 3D point cloud partition. Moreover, it facilitates visualization and storage of the points of objects in future applications. 

## 3. Edge Detection and Stixel Estimation-Based Object Segmentation

**Figure 1:**
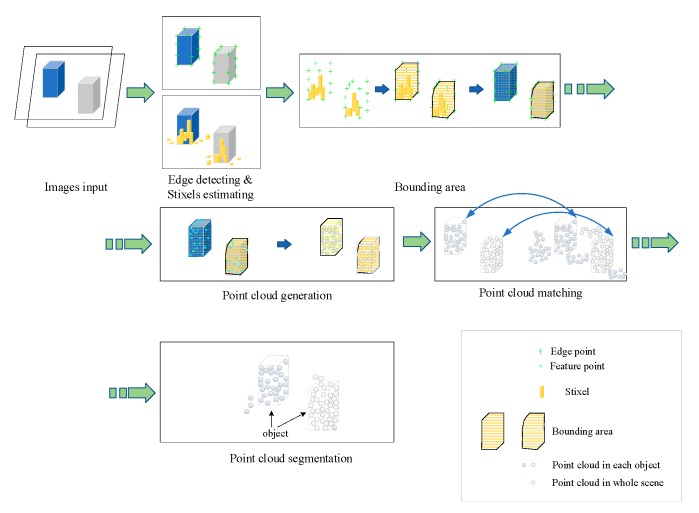
Workflow of the proposed method.

Edge detection includes manifold methods of mathematics that study to identify points in a digital image, at which the image brightness varies sharply or discontinuities [26]. The contours which are typically organized by a set of curved line segments of varied brightness can be termed as edges. The application of edge detection algorithms to images can significantly reduce the amount of data to be processed and can therefore filter out information that may be regarded as less relevant, while preserving the important structural properties of the image. If the edge detection step is successful, the subsequent task of interpreting the objects in the original image may therefore be substantially simplified. In this paper, search-based methods are applied to detect edges, followed by stereo reconstruction in detected edge areas. Then, the sparse point clouds of objects are obtained and dense point clouds are matched. Finally, the point clouds of objects are partitioned to represent the 3D objects in driving environments. Figure 1 illustrates the proposed workflow of the edge detection and stixel estimation-based segmentation method in 3D point clouds.

To satisfy the real-time and accuracy in this paper, the edge detection and stixel estimation-based method is proposed to implement efficient segmentation of 3D point clouds. The performance of the proposed method depends on edge detection, and fast edge detection methods are acknowledged as wide-used methods with good performance. Therefore, this paper takes advantage of a fast edge detector [27] to detect the object contour. Finally, we partition the 3D point cloud of the specific object according to the detected contour. The steps of the proposed method are as shown in Algorithm 1.

**Algorithm 1** Edge- and Stixel-Oriented 3D Point Cloud Segmentation
Capture the corresponding images $image_{left}$, $image_{right}$ from the stereo camera.Detect the edges $\left\{e_{l}^{1},e_{l}^{2},\ldots ,e_{l}^{n},e_{r}^{1},e_{r}^{2},\ldots ,e_{r}^{m}|n,m\in N\right\}$ using the structured forests-based edge detector.Generate the edge candidate points $\left\{c_{l}^{1},c_{l}^{2},\ldots ,c_{l}^{p},c_{r}^{1},c_{r}^{2},\ldots ,c_{r}^{q}|p,q\in N\right\}$ according to the rules given in the following Section 3.1.Identify the utmost 4 bounding areas $\left\{b_{l}^{1},b_{l}^{2},b_{l}^{3},b_{l}^{4},b_{r}^{1},b_{r}^{2},b_{r}^{3},b_{r}^{4}\right\}$ in each image based on the edge candidate points.Generate the utmost 4 sparse point clouds (SPCs) $\left\{SPC_{1},SPC_{2},SPC_{3},SPC_{4}\right\}$ of objects in the bounding areas.Match the sparse point cloud $SPC_{i}$ with the dense point cloud DPC.Segment the dense point cloud $DPC_{j}$ of objects from the sensed dense point cloud DPC.


### 3.1. Edge Detection, Stixel Estimation, and Boundary Area Selection

In this work, the goal of edge detection is to determine whether an object exists in an image. Instead of searching for an object at every image location and scale, a set of object bounding box proposals is proposed with the goal of reducing the set of positions that need to be further analyzed. Edge detection works as an object proposal generator to improve the recall and efficiency.

**Figure 2:**
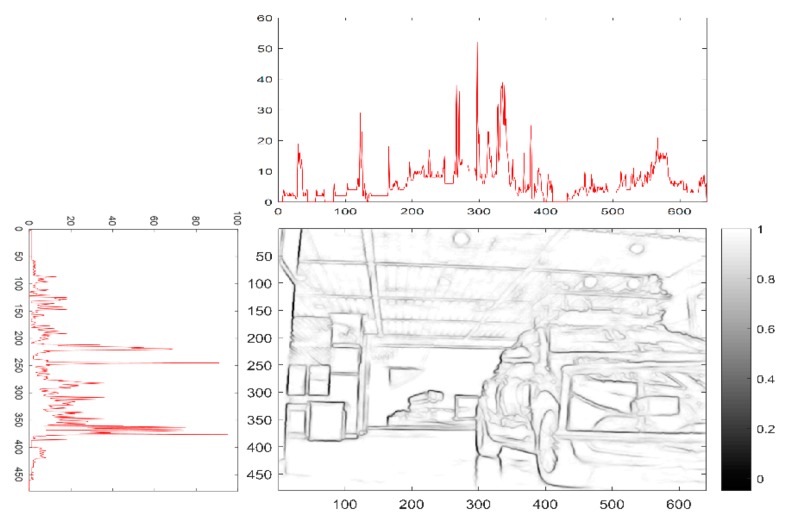
Frequency histograms of edge binary image in horizontal and vertical directions.

The structured forest-based edge detector is an outstanding performer to extract useful structural information from different visual objects. It dramatically reduces the amount of data to be processed, because edge detection can select meaningful regions rather than the whole image. Among the edge detection methods developed so far, the structured forest-based edge detection algorithm is one of the most strictly defined methods that provides good and reliable detection. Most vehicles’ rear-view shows horizontal and vertical edges, and it may be useful for bounding area generation. A group of horizontal and vertical edges that form a rectangular shape with an aspect ratio between 0.4 and 1.6 are good candidates for potential vehicles [28]. The clue has been employed to locate the position of a vehicle after initial ROI (Region of Interesting) was found based on the cue using the shadow underneath the vehicle [29]. In this work, the object proposals are given by edge box and stixel estimation, as shown in Figure 2. Similar to the v-disparity image, the frequency histograms of the edge detector are detected from the horizontal and vertical directions, respectively. In the binary image, we can find that there are more contour lines over the objects, and the values of frequency in the histograms are higher in the corresponding parts. Combined with stixel estimation, the double thresholds are deployed for object proposal, which allows the location of the object to be more accurately determined.

The process of the edge detection-based method deployed the structured forest based-detector to find the edge in this paper, and then constituted a closed region for 3D segmentation to form a semantic segmentation in the 3D point cloud. As the Algorithm 2 shown, the edge detection-based algorithm can be divided into six steps.

**Algorithm 2** Edge Detection and Stixel Estimation-Based Segmentation
Use $y\in Y={\mathbb{Z}}^{d\times d}$ to denote the segmentation mask, use $y^{\prime }\in Y^{\prime }={\left\{0,1\right\}}^{d\times d}$ for a binary edge map.Find the intensity gradients of the image. Generate candidate features x(i,j,k).Define a mapping Π:Y→Z.Run the structured edge detector on the original, half, and double resolution version of I and average the result of the three edge maps after resizing to the original image dimensions.Track the edges by hysteresis thresholding: Finalize the detection of the edges by suppressing all the other edges that are weak and not connected to strong edges.
$$\begin{array}{l} H_{ij}=\frac{1}{2\pi \sigma^{2}}\exp\left(-\frac{{\left(i-\left(k+1\right)\right)}^{2}+{\left(j-\left(k+1\right)\right)}^{2}}{2\sigma^{2}}\right);\\ 1\leq i,j\leq \left(2k+1\right) \end{array}$$Generate bounding area of candidate points.
$$C_{horizon}=\sum_{i}^{x}\frac{1}{2\pi \sigma^{2}}\exp\left(-\frac{{\left(i-\left(k+1\right)\right)}^{2}+{\left(j-\left(k+1\right)\right)}^{2}}{2\sigma^{2}}\right)*G_{x};1\leq i,j\leq \left(2k+1\right)$$
$$C_{vertical}=\sum_{j}^{y}\frac{1}{2\pi \sigma^{2}}\exp\left(-\frac{{\left(i-\left(k+1\right)\right)}^{2}+{\left(j-\left(k+1\right)\right)}^{2}}{2\sigma^{2}}\right)*G_{y};1\leq i,j\leq \left(2k+1\right)$$
where x,y are the coordinates of pixel, σ is the standard deviation of the Gaussian distribution, I is the matrix of the image, and $C_{horizon}$, $C_{vertical}$ are the horizontal and vertical direction values of the histograms in Figure 2.


The edge points are detected in the corresponding images, and the edge points of objects are more than the background. The region where the value of edged histograms is higher than the other regions can be regarded as the area that objects are located. We found the candidate point of bounding area near the area of high histogram value.

**Figure 3:**
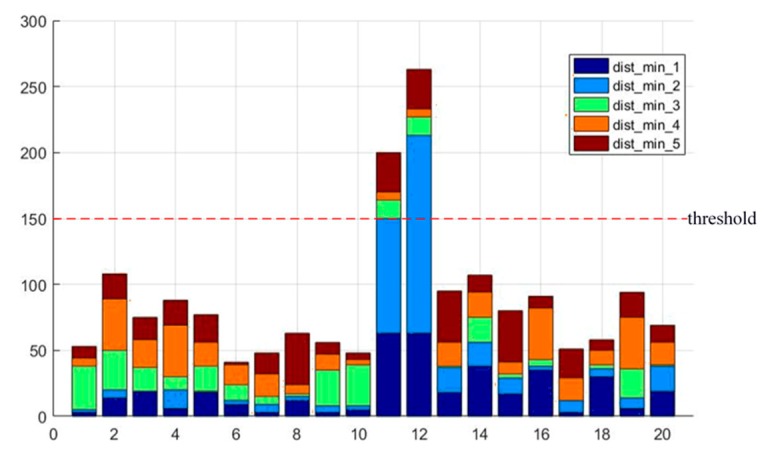
The five shortest distances from 20 candidate edge points.

**Figure 4:**
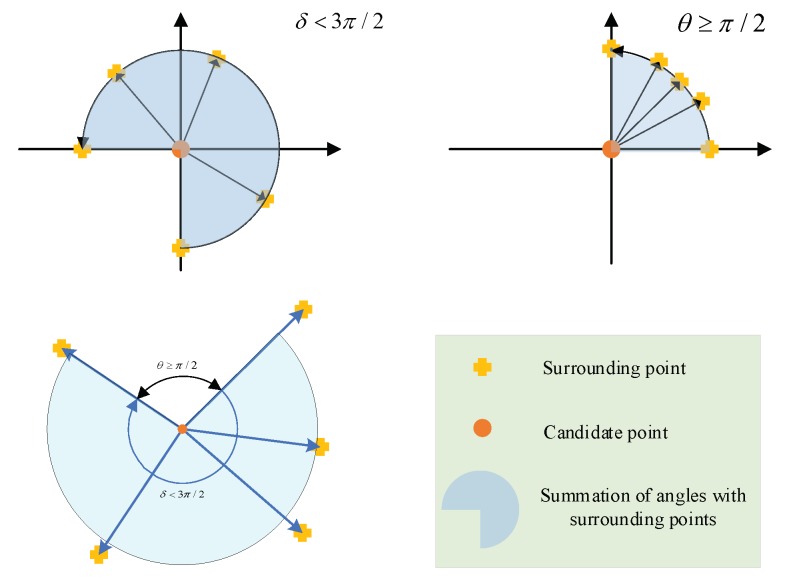
Summation of the angles of the five surrounding points.

Candidate edge points are then verified by comparing the distance of the candidate edge points with other points around the candidate edge points. According to the Lampert assumption [30], compared with the background, there are more edge points on the objects. As Figure 3 shows, if the five adjacent candidate edge points of shortest distance exceed the threshold, this candidate point will be ignored, which means the point cannot be the edge point. If the five adjacent candidate edge points of shortest distance do not exceed the threshold, then they are judged with the following rules. As shown in Figure 4, if the accumulated angle summation of the five points around the candidate edge point does not exceed 3π/2, the candidate point can be determined as the edge point according to the non-maximum suppression principle of the canny edge detector. The algorithms of edge point determination are stated in Algorithm 3.

**Algorithm 3** Determining the Edge Points from Candidate Edge Points**Input: Candidate Edge Points**$P_{n}(0<n<N)$ **Output: Edge Points**$P_{m}$(0<m<M) $While(P_{n}\neq \phi )$   If$P_{n}(0<n<N)$∈**Candidate Edge Points**    If$distance\left(P_{n}\right)<threshold$       $acc\_arc=\sum_{i=1}^{5}arc\left(P_{n}\cdot P_{i}\right)$, $P_{i}$ is the 5 nearest candidate edge points.            If
acc_arc<threshold             $P_{m}$ = $P_{n}$             m=m+1,
n=n+1           $\begin{array}{cc} end & if \end{array}$    $\begin{array}{cc} end & if \end{array}$   $\begin{array}{cc} end & if \end{array}$ $\begin{array}{cc} end & while \end{array}$


Then, we deployed a stixel estimation method to locate the objects as the double threshold with edge proposal. As shown in Algorithm 4, the stixel estimation method can be divided into four steps:

**Algorithm 4** Algorithm of Stixels Estimation
A pixel-wise cost volume $C_{ijk}={\left\|p_{ij}-p_{i\left(j-k+2\right)}\right\|}_{1}$ is computed from the input rectified stereo images;Generating the v-disparity image and detecting the ground plane on it;Generating the cost matrices $c_{o}=\sum_{v(k)}c\left(i,j,k\right)$, $c_{g}=\sum_{\left|V\right|}c\left(i,j,f_{ground}\left(k\right)\right),v\left(k\right)=f_{ground}^{-1}\left(k\right)$;The estimated stixel disparities are used to estimate the stixel heights $c_{h}=\sum_{v\left(k\right)}\left|c_{o}-1\right|+\sum_{v\left(k\right)}\left|c_{o}+1\right|$.


**Figure 5:**
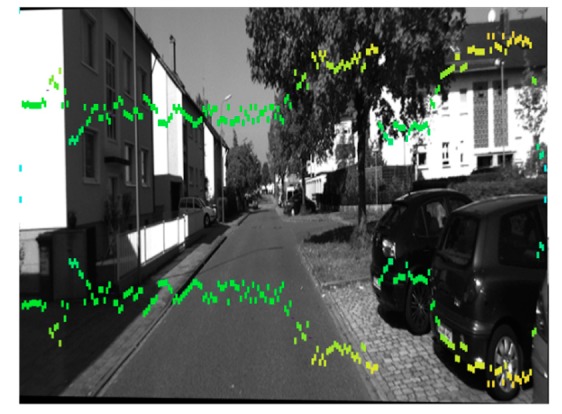
Stixel estimation in a country road.

As shown in Figure 5, the objects in a country road are surrounded by the estimated stixels. 

The location of edge points can be ascertained by the edge detection and stixel estimation, and the uncertainty of the edge points can be reflected by covariance estimation, and measured by the inverse of Hessian matrix:

(1) $$\Sigma^{i}={\left[\begin{array}{cc} D_{xx}\left(p,\sigma_{i}\right) & D_{xy}\left(p,\sigma_{i}\right)\\ D_{xy}\left(p,\sigma_{i}\right) & D_{yy}\left(p,\sigma_{i}\right) \end{array}\right]}^{-1}$$

where $D_{xx}$, $D_{xy}$, and $D_{yy}$ are the second derivative of $D\left(p,\sigma_{i}\right)$, and $p={\left(x,y\right)}^{T}$ is the location of the edge point on $\sigma_{i}$. Besides, we estimated the transform matrix $T_{i}$, $T_{j}$ from continuous images by tracking the edge points and estimated stixels, respectively. Moreover, we deployed bundle adjustment to minimize the reprojection error to determine the location of the objects.

(2) $$E=\sum_{i}\left(P_{ci}-\left(r_{i}P_{pi}+t_{i}\right)\right)+\sum_{j}\left(P_{cj}-\left(r_{j}P_{pj}+t_{j}\right)\right)$$

where $P_{ci}$, $P_{cj}$ are the edge points and stixel points in the current frame, respectively, and $P_{pi}$, $P_{pj}$ are the projection points of edge points and stixel points in next frame. The transformation matrix $T_{i}=\left[\begin{array}{cc} r_{i} & t_{i} \end{array}\right]$, $T_{j}=\left[\begin{array}{cc} r_{j} & t_{j} \end{array}\right]$. We used the spatial cue, stixel estimation and temporal cue, and optimized transformation matrix to locate the objects.

**Figure 6:**
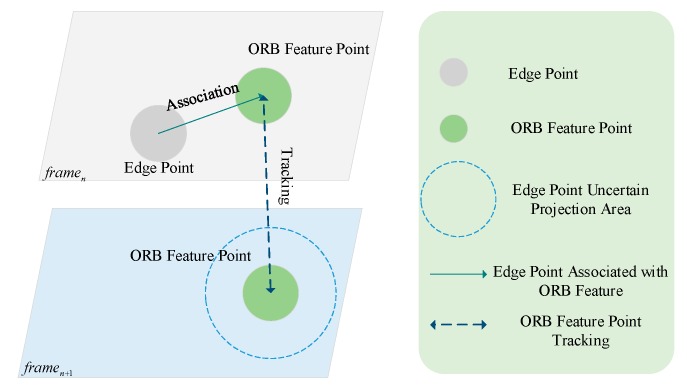
Edge point tracking and optimizing strategy.

**Figure 7:**
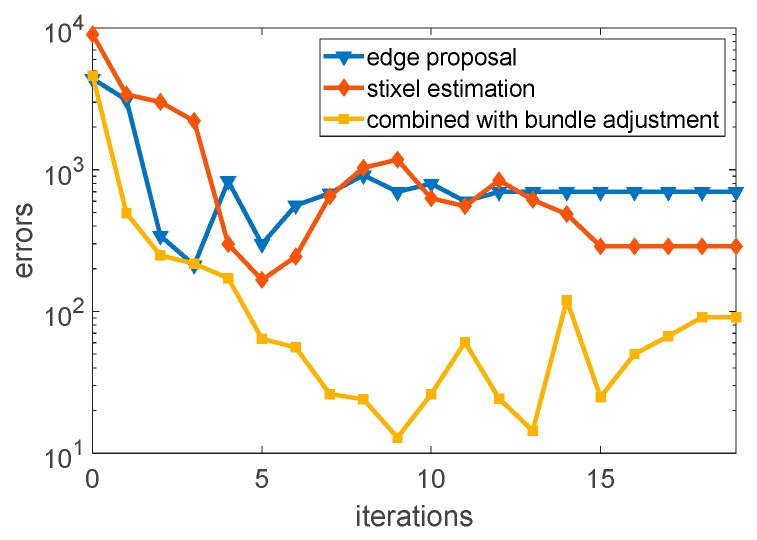
Edge point tracking without/with optimizing.

As shown in Figure 6, the edge points were tracked by using the nearest ORB (Oriented FAST and Rotated BRIEF) feature points, due to the edge points without feature descriptors. Therefore, we deployed the ORB feature points to track the edge points from $frame_{n}$ to $frame_{n+1}$, and optimize the transformation matrix by using bundle adjustment. The results of the combined method optimized by bundle adjustment are shown in Figure 7.

**Figure 8:**
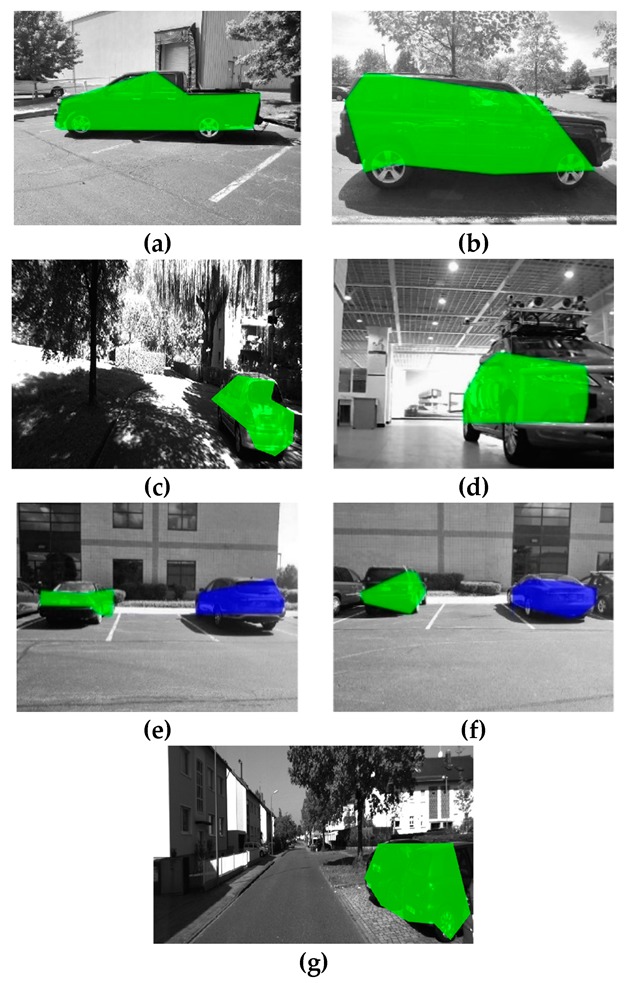
Bounding area in 2D images based on edge points in different scenarios. (**a**) outdoor parking lot, single object; (**b**) outdoor parking lot, single object; (**c**) weak light, single object; (**d**) indoor parking lot, single object; (**e**) outdoor parking lot, multi objects; (**f**) outdoor parking lot, multi objects; (**g**) roadside, single object.

After calculating the angle of the surrounding points, the candidate edge point can be verified as the edge point. The remaining edge points are connected to form a polygonal bounding area. The object is deemed to be covered by the bounding area. As shown in Figure 8, Figure 8a–d shows the scenarios with a single object, of which Figure 8d is the scenario of an indoor parking lot and Figure 8a,b shows the scenarios of an outdoor parking lot, and Figure 8c is the scenario that objects are in weak light. Furthermore, Figure 8e,f shows the scenarios with two objects, and Figure 8c,g shows the scenarios on a country road from KITTI. The green and blue polygon regions are the bounding areas.

### 3.2. 3D Object Reconstruction with 2D Image Pairs

The detected edge and estimated stixels provide a bounding area for feature detection in 2D image pairs. The image features are detected in the bounding area. In the bounding area, we take advantage of the good performance of the Speed Up Robust Features (SURF) operator in image transformations to detect the feature points. The feature points in the corresponding image pairs are stereo matched to reconstruct these objectives in a 3D coordinate system. Based on the previous section, we consider the bounding areas covered on the objects. Then, the objects are reconstructed with corresponding images in these bounding areas, and we use the 3D point cloud of these objects to match the wide-angle point cloud. Finally, the objects in dense point cloud are segmented. The next section describes the matching and segmenting of objects in detail.

**Figure 9:**
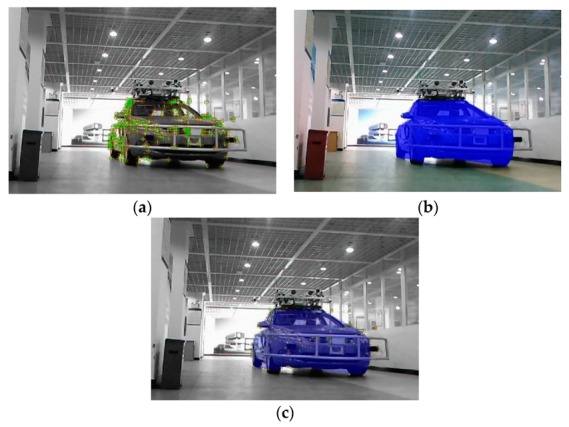
Feature detection in the object area. (**a**) matched points in bounding area; (**b**) bounding area; (**c**) superimposed area of (**a**,**c**).

As Figure 9 shows, Figure 9a is the matched points in the bounding area based on edge detection in 2D corresponding images. The blue shadow area in Figure 9b is the bounding area according to the edge points. Figure 9c is the superimposed area of Figure 9a,b. This step helps to reconstruct the 3D point cloud of an object in an interesting area.

### 3.3. 3D Point Cloud Matching and Segmentation

In this paper, our aim is to segment objects accurately and rapidly from the whole scene point cloud. We only match the 3D point cloud of objects with the whole scene point cloud. In this step, the norms of the point cloud of an object are matched with norms of the whole scene point cloud. It signifies that the reconstructed point cloud of an object can match the exact location directly in the whole scene point cloud. Therefore, the real location of objects can be found in the real running environment veritably. The matched point clouds in the whole scene point cloud remain, and the rest of the points are removed. The remaining point clouds represent the objects we want to segment. The details of 3D point cloud matching and segmentation will be given in the following subsections.

#### 3.3.1. 3D Point Cloud Matching

We get the reconstructed objects in the bounding areas from Section 3.2 to obtain the norms $\left\{n_{1},n_{2},\ldots n_{O},O\in N\right\}$ of the 3D point cloud of this reconstructed object. The 3D points in the sparse point cloud are reconstructed by 2D image feature points. According to the method of perspective n points, the location of the reconstructed points of an object in the whole scene point cloud can be calculated according the number of frames and the transformation matrix. The point cloud of an object can be located in the whole scene point cloud according to the frame number, after registering the spare point cloud in the whole scene point cloud. The norms are matched with the norms $\left\{n_{w1},n_{w2},\ldots n_{wP},P\in N\right\}$ of the whole scene point cloud by calculating the summation of distance, $distance_{sum}$. The area of objects in the point cloud of the whole scene are ascertained according to the $distance_{sum}$ of norms. If the $distance_{sum}$ is less than the threshold ε<1000, the point cloud of objects is matched, otherwise the objects are not regarded in the scene.

**Figure 10:**
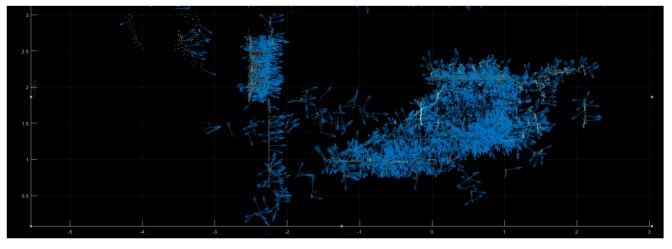
Estimated norms of the point cloud without segmentation.

As Figure 10 shows, the norms of objects in the bounding areas are matched with the point cloud of the whole scene. Then, the 3D point cloud will be segmented as per the following statement.

#### 3.3.2. 3D Point Cloud Partition

First the center point of the sparse point cloud is calculated, then the points are projected to the x−o−y plane, and the minimum rectangle enclosure of the objects in the 2D plane are generated. The heights of objects are from the sparse point cloud of objects. Thus, the 3D bounding boxes of objects are generated. If the $\sum_{i}distance_{sum}^{i}$ of the location between the point cloud of objects and the whole scene point cloud are lower than the threshold Δ<1000, the 3D bounding box are drawn in the whole scene point cloud according to the location of center point. The points in the 3D bounding box located in whole scene can remain. Then, obtain the limit points in the x, y, z axes, respectively. We partition the object point clouds along the z axis into several layers at every 10 points, thus the contour profile of objects is ascertained by compounding the limit points and the edge points. The rest of points, which are regarded as the background, are deleted. We get the partitioned point cloud of objects from the point cloud of the whole scene.

**Figure 11:**
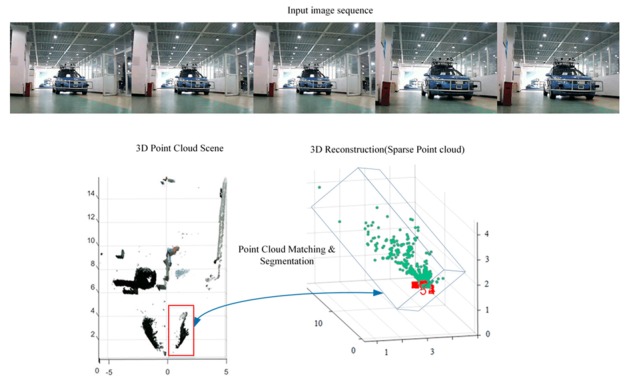
Point cloud matching and segmentation.

**Figure 12:**
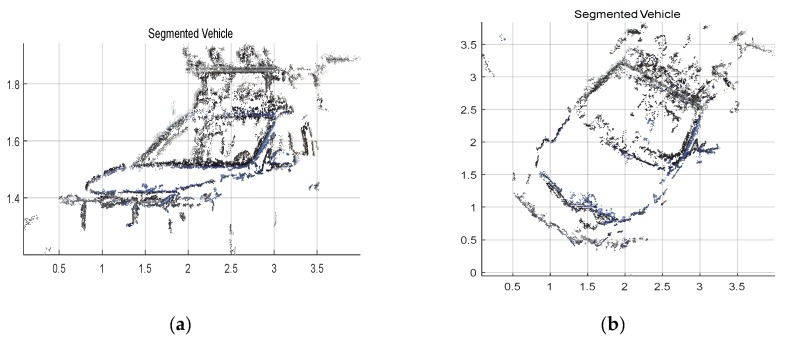
Segmented vehicle in a 3D point cloud with front and top view. (**a**) 3D point cloud of segmented vehicle from front view; (**b**) 3D point cloud of segmented vehicle from top view.

We can find out from Figure 11 that the input is image sequence. The sparse point cloud of objects according to the algorithms which are stated in this section are gained, and the sparse point cloud is matched with the dense point cloud of the whole scene. We set the maximum values of the sparse point cloud in the x,y,z axes, respectively, and then, the values form a bounding box in 3D point cloud. At last, the points in the bounding box remain and the rest of the points are removed. Thus, the result of segmenting the objects from the whole scene 3D point cloud has been implemented. As shown in Figure 12, the point cloud of the object is segmented. Figure 12a is the front view and Figure 12b is the top view. According to the coordinate values in Figure 11 and Figure 12, the volume of the point cloud is reduced and the points are also reduced. The objects can be detected and segmented accurately and quickly.

## 4. Experimental Result

We tested the proposed algorithm on two types of datasets, one of which was the dataset of the point cloud that was generated from stereo camera mounted on our experiment vehicle. The stereo camera was built up by two monocular cameras (Basler ace-acA2500-60uc), and the baseline was 60 cm. The number of image pairs was 385, and the size of the dataset was greater than 1 GB. The image size was 1920×1080 (this dataset can be download from https://pan.baidu.com/s/18JNburRaGKSVlrOq9ARy1A#list/path=%2F, password: 66ib). We acquired these images in the campus scene and country road. The other was downloaded from the website of KITTI (a project of Karlsruhe Institute of Technology and Toyota Technological Institute at Chicago, IL, USA) [31]. The number of pairs was 200. The size of the dataset was 320 MB, and the image size was 1242×375. The downloaded dataset only provided stereo images for training and testing of segmentation. The dataset from KITTI did not provide the stereo-generated point cloud, we just tested the segmentation in 2D images. In this paper, we tested the performance of segmentation with different algorithms, and we compared several performance criterions, including F-measure with different scale of data, F-measure with different algorithms, accuracy and the number of points after segmentation. We deployed the semantic image as the ground truth of segmentation. The segmented pixels were projected in one class of semantics and if the IoU (Intersection over Union) was greater than 0.5, we regarded them as true positives. There were four scenarios in our own datasets: The first scenario only contained one vehicle; the second scenario contained one vehicle and one pedestrian; the third scenario contained two vehicles; and the last scenario contained two vehicles and one pedestrian. In these test scenarios, the boxes represented the fixed objects, and the pedestrian represented the moving object.

To present a quantitative evaluation of the proposed method, we employed three criteria [32]:

$$\mathrm{Precision}=\frac{\left|GT\cap DR\right|}{\left|DR\right|}$$

$$\mathrm{Recall}=\frac{\left|GT\cap DR\right|}{\left|GT\right|}$$

$$\text{F-measure}=\frac{2\times \mathrm{Recall}\times \mathrm{Precision}}{\mathrm{Recall}+\mathrm{Precision}}$$

where GT represents the set of pixels that are classified to a specific category by the proposed method, and DR represents the set of pixels that are manually labeled to a specific category (i.e., ground truth). F-measure is the weighted harmonic mean of Precision and Recall, which is used to quantify the overall performance of the segmentation.

**Figure 13:**
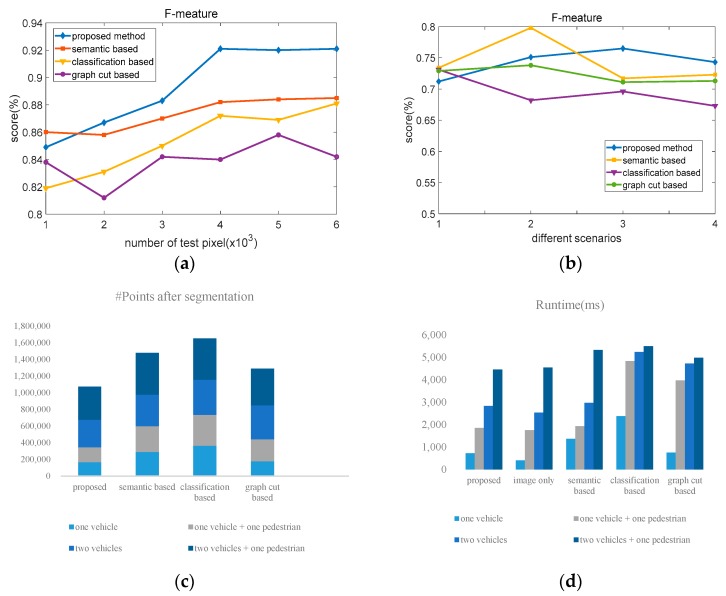
Experiment results with different algorithms. (**a**) F-measure of four algorithms with different data scales; (**b**) F-measure of four different algorithms in four scenarios; (**c**) number of points after segmenting with four different algorithms in four scenarios; (**d**) runtime of four different algorithms in four scenarios.

Figure 13a illustrates the performance of four different methods with different data scales. It shows that the proposed method maintains exceptionally high performance when the data scales is close to 4000. As compared with other three methods, the average of the proposed method is 4.48% higher than that of the other three methods. Figure 13b shows the performance of four different methods. It indicates that all methods can most accurately segment the objects in the simplest scenario where there is only one box. The performance of these methods decreases with the increase of the scenario’s complexity. Moreover, results of the proposed algorithm are 3.2%, 1.5%, and 1.1% higher than the other three methods, respectively. Table 1 and Table 2 compares the precision and recall of the four different methods, respectively. 

In the stage of point cloud segmentation, Figure 13c shows the number of points after the objects are segmented from the point cloud of the whole scene. It shows that the proposed method is able to represent the same objects with a fewer number of points. The average number of points in the proposed method is 37.3% less than the other three methods. Figure 13d explains the computation time of the four methods with four experimental scenarios. The proposed method consumes the least time to segment the same objects, which is helpful for real-time visualization and path planning. From the figures and tables, we can find that the accuracy and real-time satisfy the requirement of autonomous vehicles.

**Figure 14:**
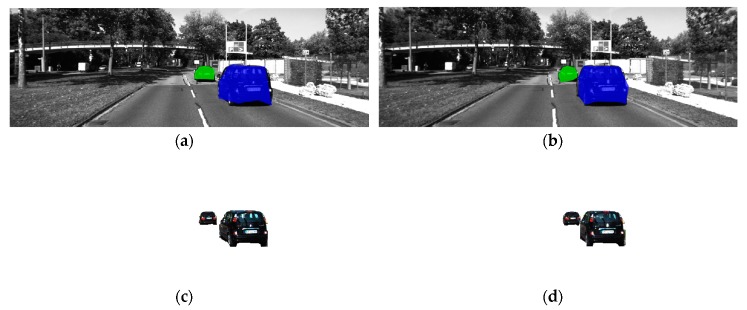
Object segmentation in corresponding image pairs. (**a**) Bounding area of objects determined in left view image; (**b**) bounding area of objects determined in right view image; (**c**) object segmentation in the left view image; (**d**) object segmentation in the right view image.

**Figure 15:**
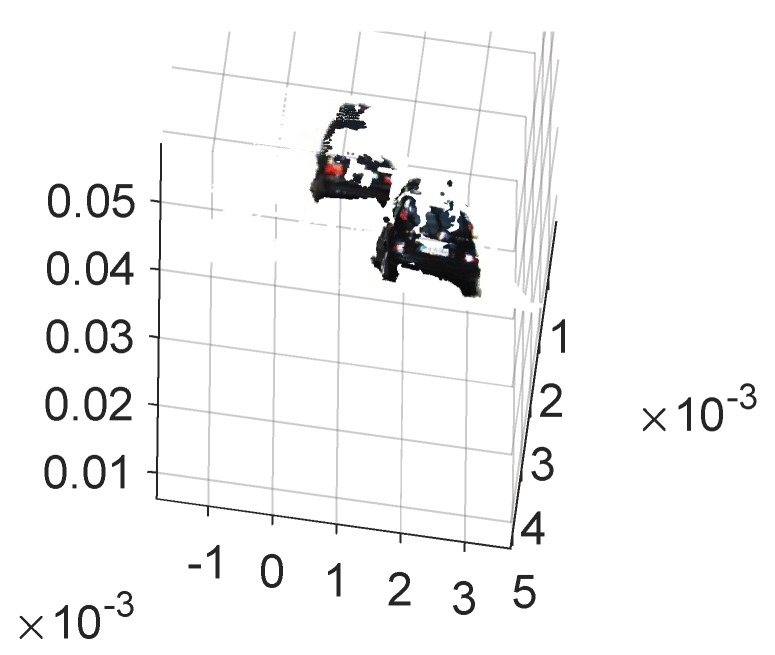
Object reconstruction according to the bounding area.

As shown in Figure 14, taking a scene in KITTI as the example, the objects are segmented according to the determined bounding area from the corresponding image pair, followed by Figure 15, in which the objects are reconstructed locally without other elements. Therefore, the objects can be located in 3D world coordinates. The availability of the proposed method is verified for the application in 3D object detection for autonomous vehicles.

**Figure 16:**
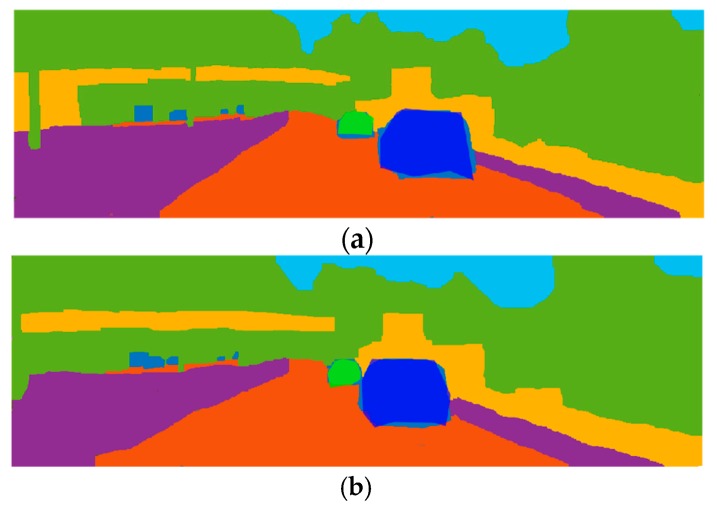
Ground truth semantic segmentation and bounding area. (**a**) Ground truth semantic segmentation-covered bounding area of objects in the left view image; (**b**) Ground truth semantic segmentation-covered bounding area of objects in the right view image.

As shown in Figure 16, the ground truth semantic segmentation images are covered by the deep blue and light green bounding area. In Figure 16a, the bounding areas cover two objects as the light green and deep blue polygons, in the center of left view image. Similarly, in Figure 16b, the two objects are covered in the right view image. The performance of the proposed method can be compared directly. Moreover, the accuracy of the proposed method can be calculated in Table 3.

As shown in Table 3, the performance of the proposed method increases 31% compared to the other three methods when the IoU is 0.6, and the accuracy is always higher than the other three. The runtime of the proposed method is approaching quasi real-time. Despite the changing of IoU, the accuracy is maintained at a high level.

**Figure 17:**
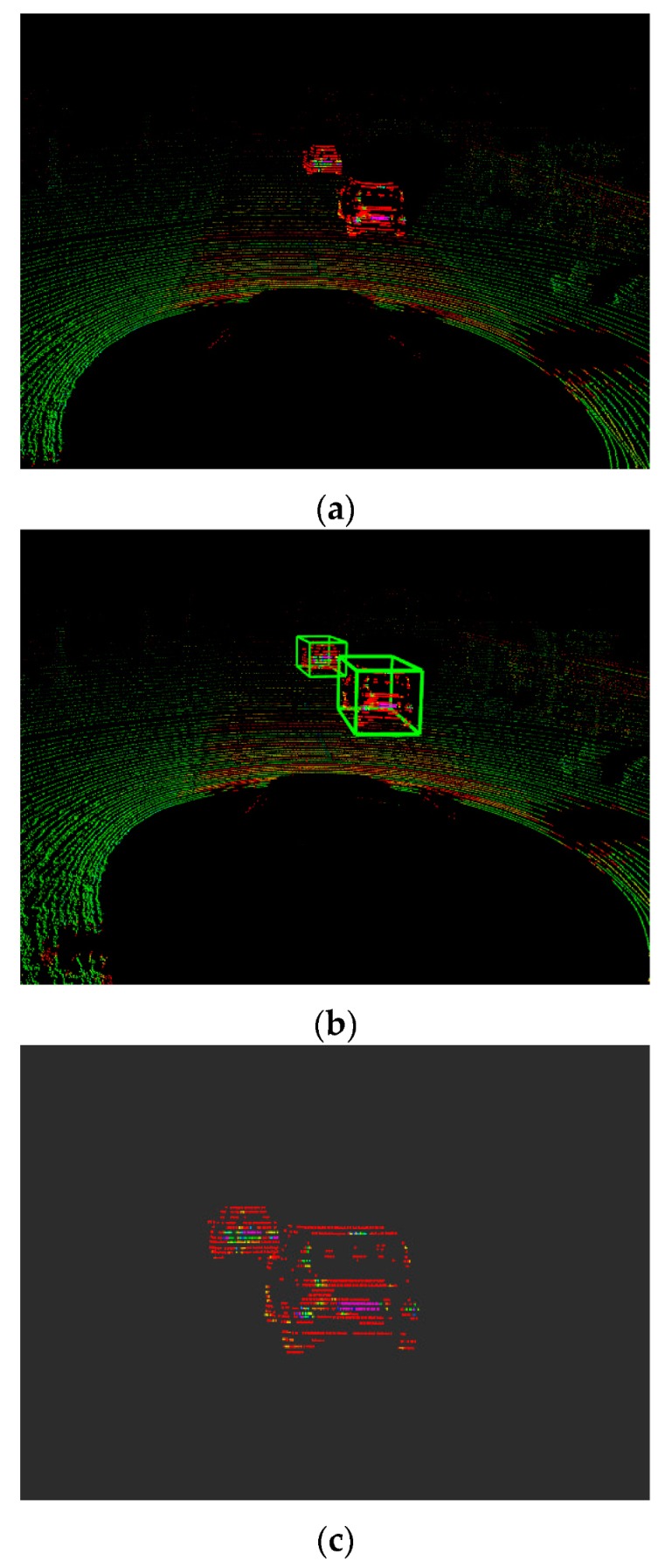
Raw data point cloud and segmented point cloud. (**a**) Raw data point cloud acquired from Lidar; (**b**) object detection in the 3D box; (**c**) object segmentation in the point cloud.

Figure 17 illustrates that the point clouds of objects are segmented from raw data point clouds from Lidar, according to the 3D coordinates of objects from Figure 15. As shown in Figure 17a, the red points almost belong to the objects in the raw data point cloud. Figure 17b shows the detected objects with the 3D bounding boxes. Figure 17c shows the segmented local point cloud of objects. The accurate point clouds of objects are segmented rapidly from whole scene point cloud.

**Figure 18:**
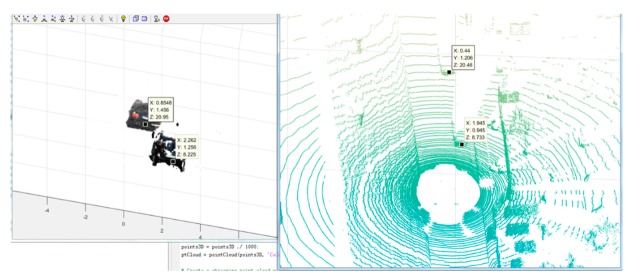
The comparison of 3D coordinates between the image 3D object point cloud and the Lidar point cloud.

Figure 18 explains that the location of the segmented 3D point cloud of objects in 3D world coordinates compared with the location of the ground truth point cloud which is generated by Lidar; the error is 0.49 m and the effective range is 50 m, so the error rate is 0.98%. The accuracy can satisfy the requirement of 3D object detection for autonomous vehicles.

## 5. Discussion

The proposed method averts the difficulty of identifying the objects directly by finding the dramatic change with edge effect and surface effect of objects to reduce computational costs and improve accuracy of objects detection. Compared with the semantic-based method, the proposed method is more time-efficient, despite the time of manual labeling. Besides, the scene of traffic is complex, and semantic-based methods are insufficient for autonomous vehicles. Similarly, the performance of classification-based methods depends on the types we gave, and classification-based methods are sensitive to scene change at a fast speed. Graph-cut-based methods easily destroy the objects, which is fatal for autonomous vehicles. After comparison with the existing methods of segmentation, the experimental results demonstrate that the proposed edge-oriented segmentation method improves the precision of 3D point cloud segmentation, and the objects can be segmented accurately. Besides, compared with the Lidar point cloud, the partitioned point cloud has a satisfied distance accuracy, which is important for autonomous vehicles, and the partitioned point cloud contains the information of color and semantics, which is helpful for object tracking. Meanwhile, the visualization of output data in ADAS (advance driving assistance system) can be greatly facilitated due to the decrease in computational time and the decrease in number of points in the object point cloud. Furthermore, the proposed method can also be available for path planning and obstacle avoidance for autonomous vehicles.

## 6. Conclusions

We propose a method for the segmentation of point clouds from a multi-view system based on edge detection. By using edge detection in 2D image pairs to initialize the bounding area of segmentation, the objects can be identified directly without hard constraints and artificial labeling. The proposed method was achieved through two main tasks. Firstly, the precision of segmentation was improved by using the bounding area in 2D image pairs, which can decrease the region in the process of 3D reconstruction. Moreover, it provides the location of objects in 3D coordinates, which helps the autonomous vehicle implement path planning. Secondly, after segmenting the dense point cloud, only the point clouds of objects are displayed and the background points are removed. It improves the performance of visualization that focuses on the location of the objects. The number of points in the point cloud is reduced due to the segmentation, which is helpful to display the point cloud of objects efficiently.

## Figures and Tables

**Table 1:** Precision of four different algorithms in four scenarios.

	One Vehicle	One Vehicle + One Pedestrian	Two Vehicles	Two Vehicles + One Pedestrian
Graph-cut-based	0.807	0.823	0.801	0.886
Semantic-based	0.813	0.731	0.735	0.775
Classification-based	0.819	0.788	0.725	0.823
Proposed	0.824	0.848	0.801	0.869

**Table 2:** Recall of four different algorithms in four scenarios.

	One Vehicle	One Vehicle + One Pedestrian	Two Vehicles	Two Vehicles + One Pedestrian
Graph-cut-based	0.781	0.796	0.686	0.723
Semantic-based	0.787	0.801	0.673	0.642
Classification-based	0.8	0.834	0.782	0.761
Proposed	0.806	0.741	0.763	0.713

**Table 3:** Accuracy measures and runtime for three Intersection over Union (IoU) metrics with four algorithms.

	IoU = 0.5		IoU = 0.6		IoU = 0.7		Runtime (ms)
	Accuracy	Recall	Accuracy	Recall	Accuracy	Recall	
Semantic-based [9]	64%	86%	36%	55%	48%	44%	1658
Classification-based [11]	58%	79%	45%	76%	62%	78%	2594
Graph-cut-based [14]	38%	69%	78%	66%	75%	65%	1583
Proposed	75%	78%	91%	84%	74%	82%	836
